# Utilize multi-metabolic parameters as determinants for prediction of skeletal muscle mass quality in elderly type2 diabetic Chinese patients

**DOI:** 10.1186/s12877-024-04827-3

**Published:** 2024-04-09

**Authors:** Huiling Chen, Jingjing Lou, Meiyuan Dong, Xintao Liu, Shijie Yan, Song Wen, Ligang Zhou, Xingdang Liu, Xinlu Yuan

**Affiliations:** 1 Department of Endocrinology, Shanghai Pudong Hospital, Fudan University Pudong Medical Center, 2800 Gongwei Road, Pudong, Shanghai, 201399 China; 2 Fudan University, Shanghai, China; 3 Department of Nuclear Medicine, Shanghai Pudong Hospital, Fudan University Pudong Medical Center, 2800 Gongwei Road, Pudong, Shanghai, 201399 China; 4 Department of General Medicine, Shanghai Pudong Hospital, Fudan University Pudong Medical Center, 2800 Gongwei Road, Pudong, Shanghai, 201399 China; 5 Shanghai Key Laboratory of Vascular Lesions Regulation and Remodeling, Shanghai, China

**Keywords:** Muscle mass, T2DM, BMI, Gender, C-peptide, Body fat, Glycemic metabolism

## Abstract

**Background:**

Sarcopenia, an age-related disorder characterized by loss of skeletal muscle mass and function, is recently recognized as a complication in elderly patients with type 2 diabetes mellitus (T2DM). Skeletal muscles play a crucial role in glycemic metabolism, utilizing around 80% of blood glucose. Accordingly, we aimed to explore the relationship between glucose metabolism and muscle mass in T2DM.

**Methods:**

We employed the AWGS 2019 criteria for diagnosing low muscle mass and 1999 World Health Organization (WHO) diabetes diagnostic standards. This study included data of 191 individuals aged 60 and above with T2DM of Shanghai Pudong Hospital from November 2021 to November 2022. Fasting C-peptide (FPCP), fasting plasma glucose (FPG), 2-hour postprandial plasma glucose (PPG) and postprandial 2-hour C-peptide (PPCP), glycated hemoglobin A1c (HbA1c), glycated albumin (GA), serum lipids spectrum, renal and hepatic function, hemoglobin, and hormone were measured. Based on the findings of univariate analysis, logistic regression and receiver operating characteristic (ROC) curves were established.

**Results:**

Participants with low muscle mass had significantly lower alanine and aspartate aminotransferase, and both FPCP and PPCP levels (*P* < 0.05). Compared with those without low muscle mass, low muscle mass group had significantly higher FPG, HbA1c, GA levels (*P* < 0.05). Body fat (BF, OR = 1.181) was an independent risk factor for low muscle mass. PPCP (OR = 0.497), BMI (OR = 0.548), and female (OR = 0.050) were identified as protective factors for low skeletal muscle. The AUC of BMI was the highest, followed by the PPCP, gender and BF (0.810, 0.675, 0.647, and 0.639, respectively), and the AUC of the combination of the above four parameters reached 0.895.

**Conclusions:**

In this cross-sectional study, BMI, Female, and PPCP associated with T2DM were protective factors for low muscle mass. BF was associated with T2DM and risk factor for low muscle mass.

## Introduction

The term “sarcopenia” was first coined by Rosenberg in 1989 to describe the condition of muscle atrophy in elderly individuals, primarily referring to the age-related decline in skeletal muscle mass [1]. Besides implement of various movements through joint contractions, muscles also serve as a major site for glucose metabolism, utilizing about 80% of overall systemic glucose by glucose transporter 4 (GLUT4), and playing a crucial role in blood glucose control [2, 3]. Research indicate that diabetes patients exhibit lower skeletal muscle index values [4], and the risk of developing muscle loss (sarcopenia) in type 2 diabetes patients is 1.5-2 times higher than in non-diabetic individuals [5]. A meta-analysis study based on Asian populations reveals that the prevalence of sarcopenia in diabetes patients is 15.9%, compared to 0.8% in non-diabetic individuals [6].. Currently, sarcopenia is recognized as a new complication of type 2 diabetes mellitus (T2DM) [7], not only leading to a decrease in the quality of life but also increasing the risk of physical disability and even death [8, 9].

In two longitudinal studies conducted in South Korea and Japan, it has been consistently demonstrated that elevated blood glucose levels, represented by HbA1c, are associated with low muscle mass in elderly diabetic patients [10, 11] Indeed, some scholars posit that skeletal muscle mass can serve as a predictive indicator for the deterioration of glucose metabolism [10, 12]. The existence of these potential correlations has indeed opened a new frontier in understanding blood glucose homeostasis in the elderly. Consequently, we focused on the glycemic status in elderly individuals with T2DM, and in order to elucidate the relationship between skeletal muscle and blood glucose metabolism.

## Materials and methods

### Study participants

**Fig. 1:**
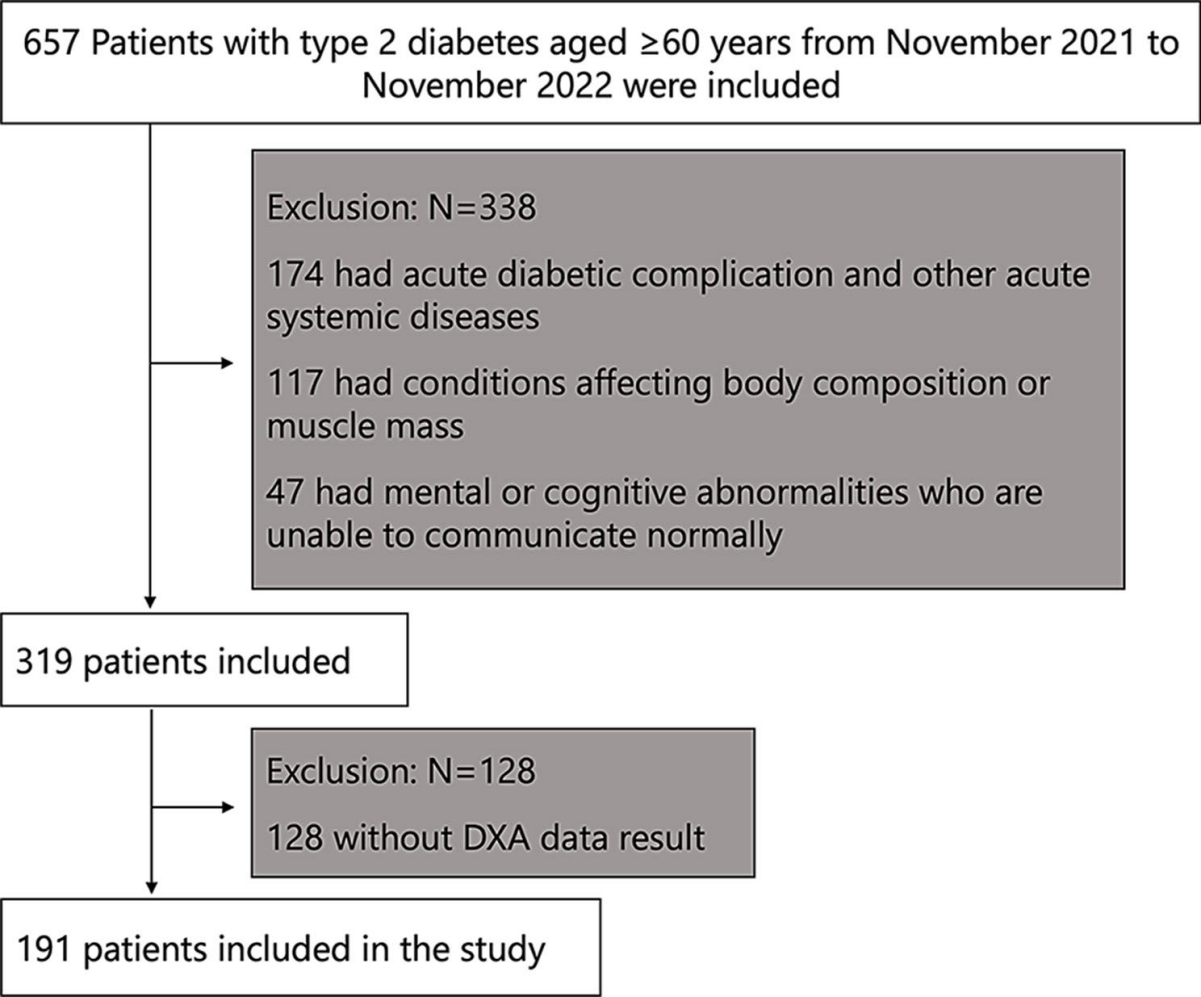
Flowchart of the participants inclusion and exclusion in the study. The diagram shows how patients were included and excluded from the target population. A total of 191 patients were finally enrolled in this study. DXA, dual-energy X-ray absorptiometry

We conducted a study on 657 Chinese elderly individuals aged 60 and above who were diagnosed with T2DM and admitted to Fudan University Shanghai Pudong Hospital between November 2021 and November 2022.The T2D diagnosis made using World Health Organization (WHO) guidelines published in 1999 [13]. Patients with acute diabetic complication and other acute systemic diseases (such as Diabetic Ketoacidosis, hyperosmolar nonketotic diabetic coma, acute hepatic insufficiency and acute renal insufficiency), conditions affecting body composition or muscle mass (Such as disability, cancer, thyroid disease, and chronic hepatic insufficiency),mental or cognitive impairment were excluded from the study, as well as the incomplete data, resulting in inclusion a total of 191 elderly patients, who were diagnosed with low muscle mass (Fig. 1).

### Assessment of low muscle mass

Muscle mass was assessed using lean mass, excluding bone mineral content (BMC), obtained from dual-energy X-ray absorptiometry (DXA) examination. In previous research, lean mass has been shown valid in represent skeletal muscle mass in the extremities [14].In our study, each participant underwent a whole-body DXA (Hologic, ASY-00409, USA) scan administered by a certified medical radiation therapist. The indicators for collection include appendicular skeletal muscle mass (ASM), upper limb skeletal muscle mass, lower limb skeletal muscle mass, trunk skeletal muscle mass, and body fat mass. According to the Asian Working Group for Sarcopenia (AWGS) consensus (2019) diagnostic criteria [15], appendicular skeletal muscle mass index( ASMI, ASM/height^2^): when men < 7.0 kg/m^2^; women < 5.7 kg/m^2^, then supported the diagnosis of low muscle mass. Upper Limb Skeletal Muscle Index (SMI-Upper) = Upper Limb Skeletal Muscle Mass (kg) / Height² (m²); Lower Limb Skeletal Muscle Index (SMI-Lower) = Lower Limb Skeletal Muscle Mass (kg) / Height² (m²); SMI-Total, total skeletal muscle index = Total Skeletal Muscle Mass (kg) / Height² (m²).

### Laboratory measurements

Following admission, professionally trained researchers will measure and gather personal information about the participants. This will include details such as BMI, age, gender, duration of diabetes, smoking and drinking history, medication history, and past medical history. Except for PPCP and PPG, which were measured using elbow venous blood 2 h after a meal, other biochemical indicators were measured using fasting elbow venous blood on the second day after admission.

HOMA-IR and HOMA-β are established metrics extensively employed in clinical practice to evaluate insulin resistance and pancreatic β-cell function in individuals with diabetes. Recognizing that some participants in this study were undergoing insulin therapy for glycemic control, we computed the HOMA index utilizing fasting C-peptide through the following formula.

HOMA index: HOMA-IR = 1.5 + fasting blood glucose × fasting C-peptide/2800; HOMA-β= × fasting C-peptide/(fasting blood glucose-3.5), (C-peptide: pmol/L; blood glucose: mmol/L) [16].

### Statistical analysis

All data were analyzed using SPSS (IBM, version 26.0) and prism (GraphPad, version 9.0). Measurement data of continuous variables are presented as the mean ± SD (standard deviation), and categorical variables are expressed as frequencies with percentages. The demographic and clinical data of the T2DM patients with and without low muscle mass were compared using the Mann-Whitney test, an independent t-test, and a chi-square test. Logistic regression was used to identify risk factors for the low muscle mass. Spearman correlation analysis was also established to identify the relationship between skeletal muscle mass and biochemical markers. Variables with statistical differences between groups were selected for the multivariate regression and spearman correlation. The level of statistical significance was set at *P* < 0.05.

## Results

### General clinical and basic laboratory characteristics

The general clinical and basic laboratory characteristics of the 191 participants with and without low muscle mass enrolled in this study are shown in Tables 1 and 2. 54 (61.4%) male and 34 (38.6%) female participants had low muscle mass, but there were no differences in age, WHR, Duration of diabetes, blood pressure, past medical history (anti-diabetic medication, insulin), Diabetic complications (Arteriosclerosis, Diabetic peripheral neuropathy, Diabetic nephropathy, or Diabetic retinopathy) between the two groups. Nevertheless, participants with low muscle mass were more likely to be male, smokers, and alcohol consumers, and they also had higher weight, Body Mass Index (BMI), Body Fat (BF), and ASMI than those without low muscle mass (*p* < 0.05). (Table 1)

**Table 1:** General clinical characteristics of the cohort that was classified according to appendicular skeletal muscle mass

	Low muscle mass *n* = 88	Normal muscle mass *n* = 103	P value
Gender(n,%)			< 0.001*
Male	54(61.4)	33(32.0)	
Female	34(38.6)	70(68.0)	
Age(years)	70.68 ± 8.06	70.58 ± 6.11	0.923
WHR	1.22 ± 0.31	1.26 ± 0.27	0.301
BMI(kg/m2)	23.78 ± 2.55	27.24 ± 3.24	< 0.001*
BF(%)	31.03 ± 7.74	34.99 ± 6.90	< 0.001*
Duration(years)	13 (8, 20)	11 (6, 1)	0.074
ASMI	5.89(5.08,6.51)	6.50(5.84,7.35)	< 0.001*
Blood pressure (mmHg)			
Systolic	129.57 ± 15.63	131.51 ± 16.18	0.406
Diastolic	76.59 ± 10.39	76.12 ± 9.42	0.776
Ever/current smoke (n,%)			0.013*
Yes	29(33.0)	18(17.5)	
No	59(67.0)	85(82.5)	
Ever/current drinking (n,%)			0.005*
Yes	18(20.5)	7(6.8)	
No	70(79.5)	96(93.2)	
History of anti-diabetic medication(n,%)			0.061
Yes	50(56.8)	72(69.9)	
No	38(43.2)	31(30.1)	
History of insulin(n,%)			0.444
Yes	37(42.0)	49(47.6)	
No	51(58.0)	54(52.4)	
History of insulin and anti-diabetic medication(n,%)			0.064
Yes	17(19.3)	32(31.1)	
No	71(80.7)	71(68.9)	
Arteriosclerosis(n,%)			0.204
Yes	82(93.2)	100(97.1)	
No	6(6.8)	3(2.9)	
Diabetic peripheral neuropathy (n,%)			0.098
Yes	69(78.4)	90(87.4)	
No	19(21.6)	13(12.6)	
Diabetic nephropathy (n,%)			0.789
Yes	41(46.6)	46(44.7)	
No	47(53.4)	57(55.3)	
Diabetic retinopathy (n,%)			0.857
Yes	11(12.5)	12(11.7)	
No	77(87.5)	91(88.3)	

WHR, waist-to-hip ratio; BMI, body mass index (weight/ height^2^); BF, body fat (%); Duration, duration of T2DM; ASMI, appendicular skeletal muscle mass index

Compared with those without low muscle mass, those with low muscle mass had lower AST and ALT(*p* < 0.05). Simultaneously, there were no differences observed between the low muscle mass group and the non-low muscle mass group in terms of HB, lipid metabolism, thyroid function, liver function, kidney function, as well as levels of vitamins and trace elements. (Table 2).

**Table 2:** Basic laboratory characteristics of the cohort that was classified according to appendicular skeletal muscle mass

	Low muscle mass *n* = 88	Normal muscle mass *n* = 103	P value
HB(g/l)	134.0(118.0,144.0)	139.0(122.5,148.0)	0.214
TG(mmol/l)	1.34 (1.03,2.00)	1.43(1.09,2.09)	0.520
CHOL(mmol/l)	4.45 ± 1.15	4.19 ± 0.93	0.092
HDL(mmol/l)	1.04(0.87,1.35)	1.04(0.87,1.23)	0.802
LDL(mmol/l)	2.74(2.16,3.49)	2.64(1.80,3.22)	0.137
FT3(pmol/l)	4.23(3.77,4.80)	4.44(4.04,4.88)	0.073
FT4(pmol/l)	15.64 ± 2.81	15.13 ± 2.45	0.187
TSH(mlU/l)	1.87 (1.32,2.95)	1.85 (1.19,2.99)	0.852
TT4(nmol/l)	87.70 (71.95,102.93)	89.70 (74.00,105.58)	0.328
TT3(nmol/l)	1.29 ± 0.30	1.38 ± 0.31	0.890
PTH(pmol/l)	4.50(3.60,6.40)	5.00 (3.90,6.85)	0.172
AST(U/L)	17.00(16.00,21.25)	20.00(16.25,25.00)	0.020*
ALT(U/L)	15.00(13.00,21.25)	19.00(14.00,27.75)	0.036*
AST/ALT	1.18(1.00,1.36)	1.04 (0.88,1.33)	0.060
ALP(U/L)	79(65,96)	73(59,86)	0.076
LDH(U/L)	179(164,198)	195(173,217)	0.083
IBILI(umol/l)	7.20(5.20,8.60)	7.00(5.20,9.30)	0.252
DBILI(umol/l)	3.40(2.50,4.90)	3.60(2.70,4.80)	0.514
TBILI(umol/l)	10.60(7.30,13.50)	11.10(7.90,14.00)	0.369
ALB(g/l)	38.40 (36.50,41.00)	38.85 (36.88,41.63)	0.622
GLO(g/l)	23.57 ± 3.34	24.19 ± 3.54	0.193
TP(g/l)	63.20 (60.70,67.50)	64.40 (59.45,70.13)	0.922
PA(mg/l)	234.00 (205.00,268.00)	247.00 (223.00,273.00)	0.060
BA(umol/l)	3.25 (2.20,5.44)	3.36 (2.14,3.36)	0.807
UA(umol/l)	292.00 (222.00,352.00)	303.00 (239.75,374.50)	0.425
eGFR	87.02(68.74,98.80)	85.09(62.98,95.32)	0.201
Cr(umol/l)	68.00(56.00,86.00)	64.00(55.50,92.50)	0.586
BUN(mmol/l)	6.60(5.30,8.20)	6.10(5.00,8.30)	0.283
Ca(mmol/l)	2.12(12.06,2.26)	2.17(2.09,2.26)	0.135
Mg(mmol/l)	0.82(0.77,0.84)	0.84(0.77,0.88)	0.103
Fe(umol/l)	11.80(9.60,15.80)	12.55(9.60,17.03)	0.129
K(mmol/l)	3.92 ± 0.41	3.93 ± 0.43	0.785
Vit B6(nmol/l)	16.53(15.74,18.13)	16.58(15.77,18.04)	0.990
Vit B9(nmol/l)	8.47(8.11,9.01)	8.48(8.14,9.10)	0.826
25-OH(nmol/l)	32.19 ± 4.42	31.62 ± 3.99	0.436
Vit E(ug/ml)	11.05(10.60,11.60)	11.03(10.59,11.38)	0.524

HB, hemoglobin; TG, triglycerides; CHOL, cholesterol; HDL, high-density lipoprotein; LDL, low-density lipoprotein; FT3, free triiodothyronine; FT4, free tetraiodothyronine; TSH, thyroid-stimulating hormone;TT3, total triiodothyronine; TT4, total tetraiodothyronine; PTH, parathyroid hormone; ALT, alanine aminotransferase; AST, aspartate aminotransferase; AST/ALT, alanine aminotransferase to aspartate aminotransferase ratio; ALP, Alkaline Posphatase; LDH, lactate dehydrogenase; IBILI, indirect bilirubin; DBILI, direct bilirubin; TBILI, total bilirubin; ALB, albumin; GLO, globulin; TP, total protein; PA, prealbumin; BA, bile acid; UA, uric acid; eGFR,estimated glomerular filtration rate; Cr, creatinine; BUN, blood urea nitrogen; Ca, calcium; Mg, magnesium; Fe, ferrum; K, potassium; Vit A, vitamin A; Vit B1, vitamin B1; Vit B2, vitamin B2; Vit B6, vitamin B6; Vit B9, vitamin B9; 25-OH, 25-hydroxy-vitamin; Vit E, vitamin E

### Characteristics of glucose metabolism

**Fig. 2:**
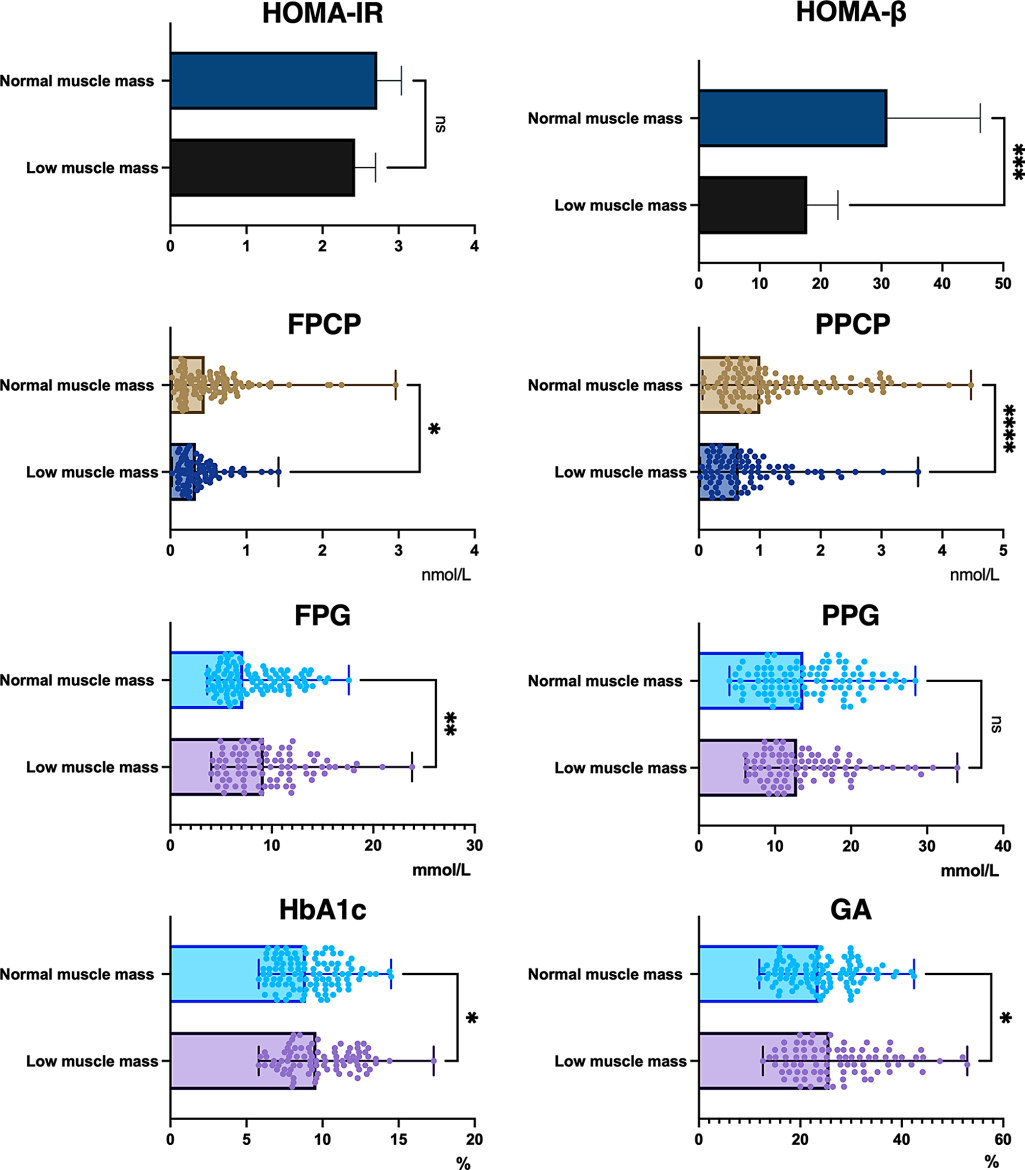
delineates the glucose metabolism characteristics in two cohorts. FPCP, fasting C-peptide; FPG, fasting plasma glucose; HbA1c, glycated hemoglobin; GA, Glycated Albumin; PPG,2-hour postprandial plasma glucose; PPCP, postprandial 2-hour C-peptide. *Note* *p < 0.05; **p < 0.01; ***p < 0.001; ****p < 0.0001

Participants with low muscle mass demonstrated notably diminished levels of HOMA-β (*P* < 0.001) and PPCP(*P* < 0.0001) in contrast to their counterparts with normal muscle mass. Moreover, FPCP was marginally lower in the low muscle mass group compared to the normal muscle mass group (*P* < 0.05). Furthermore, the low muscle mass cohort exhibited elevated levels of FPG(*P* < 0.01), HbA1c(*P* < 0.05), and GA(*P* < 0.05) relative to the normal muscle mass cohort. Regrettably, despite a slightly higher PPG and HOMA-IR in the low muscle mass group compared to the normal muscle mass group, there was no statistically significant difference between the two groups. (*P* > 0.05) **(**Fig. 2**)**.

### Associations between muscle mass and glucose metabolism in male participants with T2DM

**Fig. 3:**
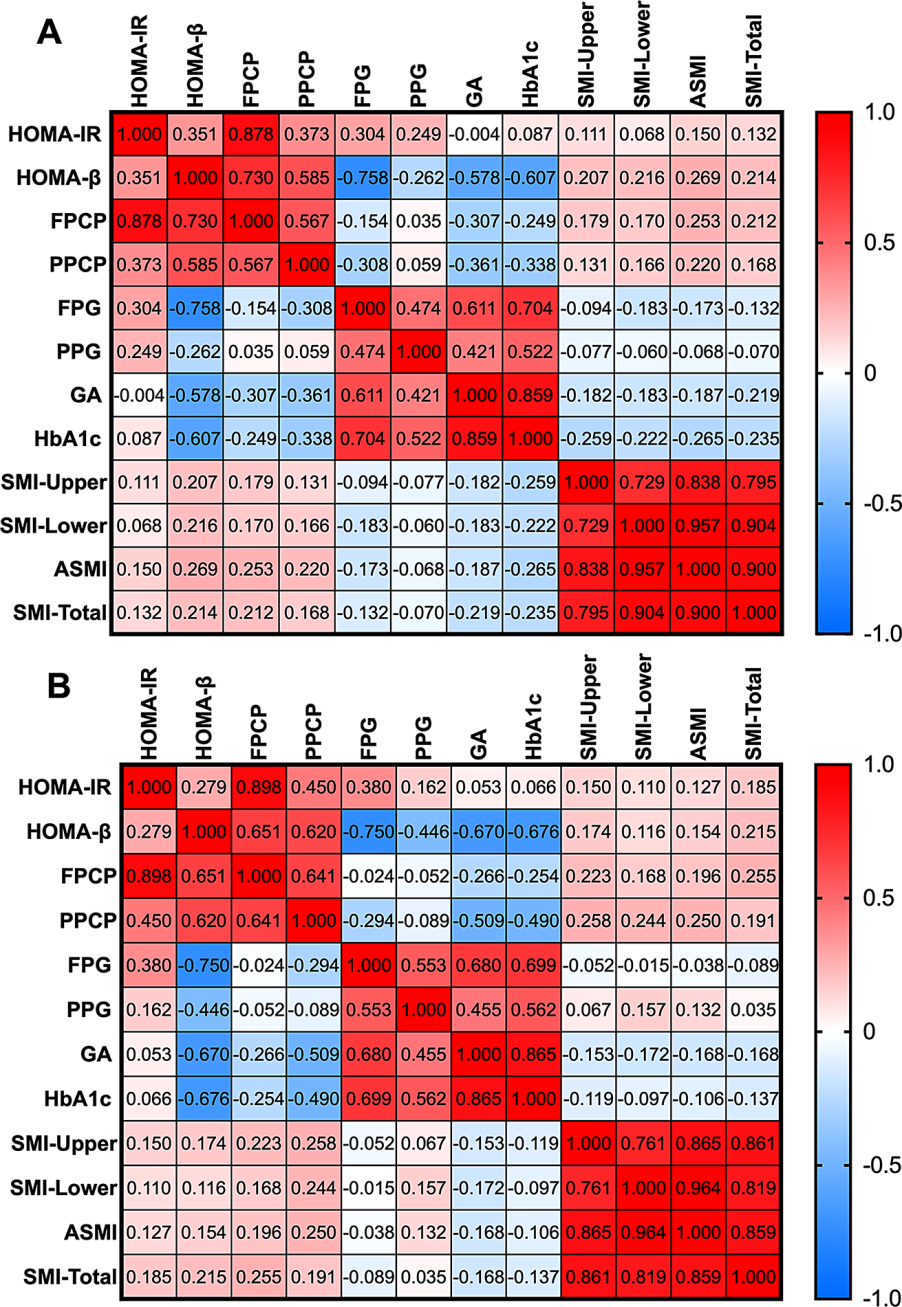
The heatmap shows the correlational relationship between the muscle mass and glucose metabolism parameters in male and female participants. The degree of the gradient in red represents the degree of positive correlation, and the blue represents the degree of negative correlation. FPCP, fasting C-peptide; PPCP, postprandial 2-hour C-peptide; FPG, fasting plasma gluco**se**; PPG,2-hour postprandial plasma glucose; GA, Glycated Albumin; HbA1c, glycated hemoglobin; SMI-Upper, upper limb skeletal muscle index; SMI-Lower, lower limb skeletal muscle index; ASMI, appendicular skeletal muscle mass index; SMI-Total, total skeletal muscle index

We performed Spearman correlation analysis to determine the correlations between muscle mass and glucose metabolism variables of T2DM. The correlation analyses between a glucose metabolism variables and muscle mass in male participants are shown in Fig. 3A.

We found that SMI-Upper (*r* = − 0.259, *P* = 0.018) and SMI-Lower (*r* = − 0.222, *P* = 0.018) was negatively correlated with HbA1c among male participants.; ASMI positively correlated with HOMA-β (*r* = 0.269, *P* = 0.013), FPCP (*r* = 0.253, *P* = 0.019), PPCP (*r* = 0.220, *P* = 0.043), but negatively correlated with HbA1c (*r* = − 0.265, *P* = 0.014) among male participants.

SMI-Total positively correlated with HOMA-β (*r* = 0.214, *P* = 0.049), but negatively correlated with HbA1c (*r* = − 0.235, *P* = 0.030) and GA (*r* = − 0.219, *P* = 0.044) among male participants.

### Associations between muscle mass and glucose metabolism in female participants with T2DM

We performed Spearman correlation analysis to determine the correlations between muscle mass and glucose metabolism variables of T2DM. The correlation analyses between a glucose metabolism variables and muscle mass in female participants are shown in Fig. 3B.

We found that SMI-Upper (*r* = 0.223, *P* = 0.023;*r* = 0.258, *P* = 0.008)and ASMI (*r* = 0.196, *P* = 0.046; *r* = 0.250, *P* = 0.010) were positively correlated with FPCP and PPCP among female participants; SMI-Lower positively correlated with PPCP(*r* = 0.244, *P* = 0.013); SMI-Total positively correlated with HOMA-β (*r* = 0.215, *P* = 0.028)and FPCP(*r* = 0.255, *P* = 0.009).

### **Logistic regression of biochemical parameters and low muscle mass**

**Fig. 4:**
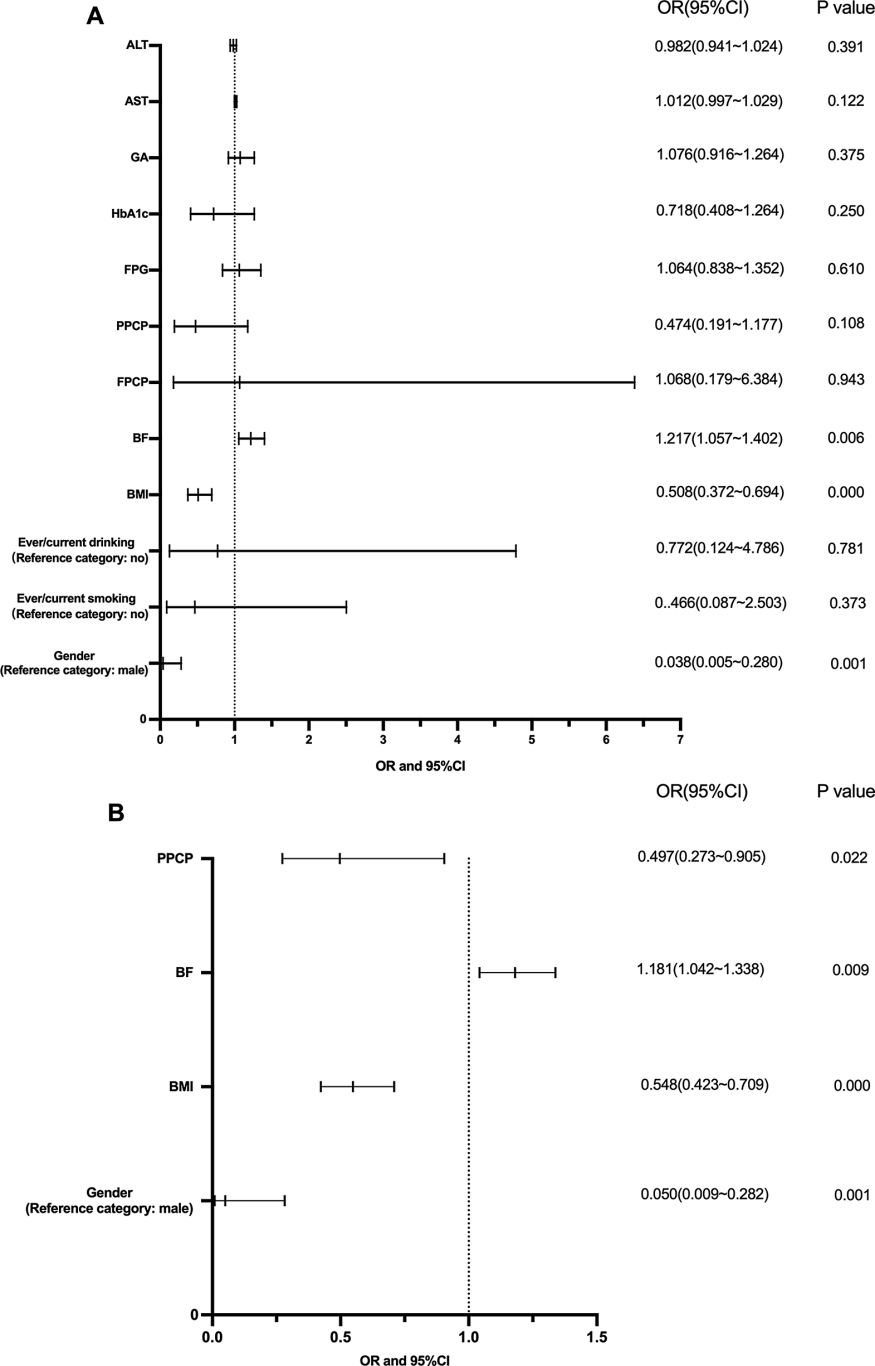
Factors associated with low muscle mass in T2DM; (**A**) Binary logistic regression analysis with stepwise backward deletion (all variables); (**B**) Binary logistic regression analysis with stepwise backward deletion (the optimal subset); GA, Glycated Albumin; HbA1c, glycated hemoglobin; FPG, fasting plasma glucose; PPCP, postprandial 2-hour C-peptide; FPCP, fasting C-peptide; BF, body fat (%); BMI, body mass index; ALT, alanine aminotransferase; AST, aspartate aminotransferase

We used binary logistic regression analysis with backward deletion to identify the optimal subset. We included all variables with statistically significant results (whose p-value is < 0.05) in the univariate analysis. (Fig. 4A) We finally got the optimal subset, we found that BF (OR = 1.181, [95% CI: 1.042–1.338] *P* = 0.009) was identified as risk factor for low skeletal muscle, BMI (OR = 0.548, [95% CI: 0.423–0.709] *P* = 0.000), PPCP(OR = 0.497, [95% CI: 0.273–0.905] *P* = 0.022) and Female (OR = 0.009, [95% CI: 0.050–0.282] *P* = 0.001) were identified as protective factors for low skeletal muscle. (Fig. 4B)

### The predictive value of parameters for muscle mass

**Fig. 5:**
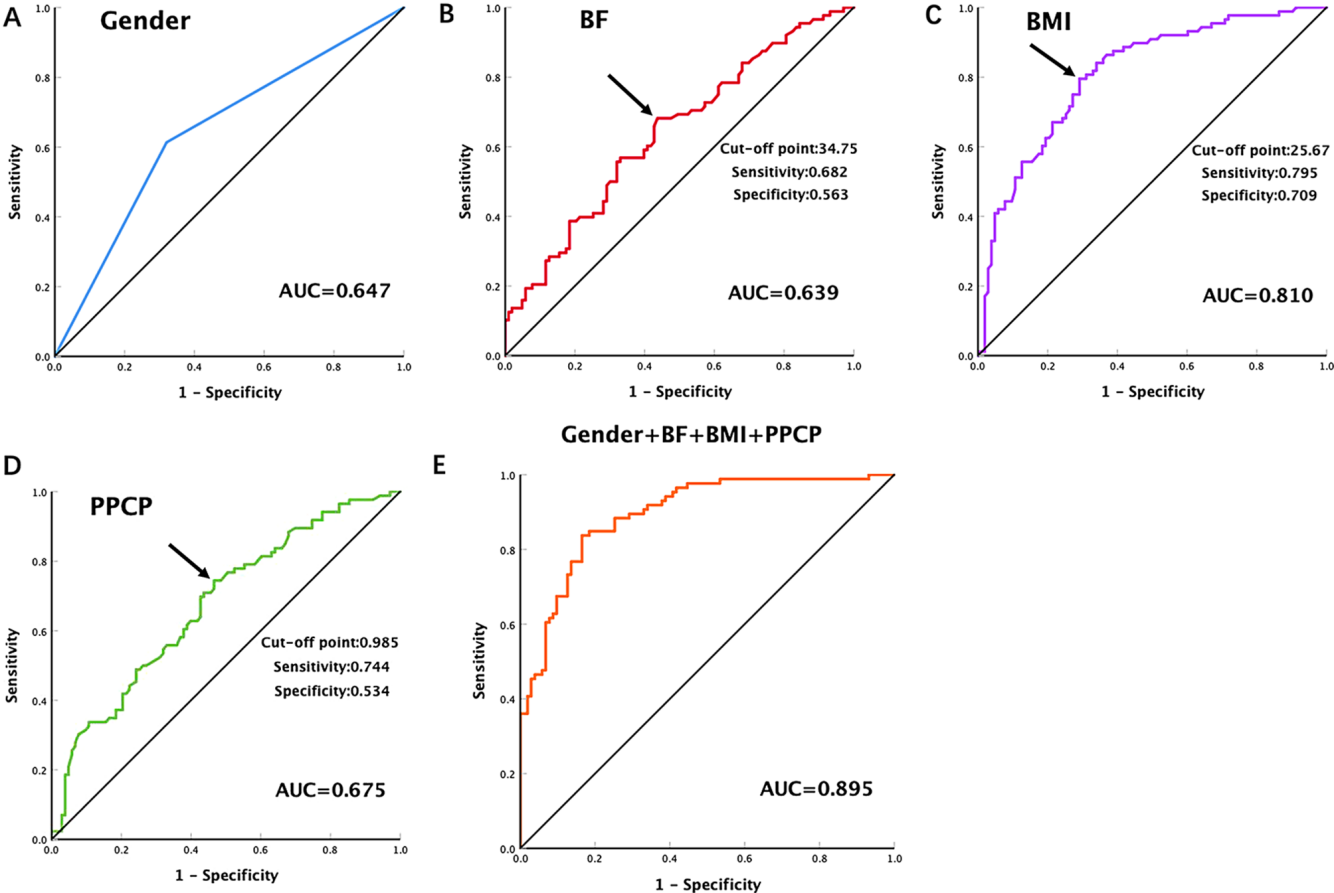
Receiver operating characteristic curves of different indicators in skeletal muscle mass. The black arrow indicates the optimal point on the ROC curve. BF, body fat (%); BMI, body mass index; PPCP, postprandial 2-hour C-peptide

We established receiver operating characteristic curves (ROC) and assessed the diagnostic performance of the indicators reflecting the low muscle mass in the Elderly T2DM cohort by the area under the curve (AUC). BMI had the best diagnostic performance AUC = 0.810 [95% CI: 0.748–0.871], followed by PPCP AUC = 0.675 [95% CI: 0.599– 0.751], gender AUC = 0.647 [95% CI: 0.568–0.725], and BF AUC = 0.639 [95% CI: 0.561–0.717]. The result of optimal cut-off point calculated by Youden Index. Cut-off point, sensitivity, specificity and Youden index of BF are 34.75, 68.2%, 56.3% and 24.5%, respectively.; Cut-off point, sensitivity, specificity and Youden index of BMI are 25.67, 79.5%, 70.9% and 50.4%, respectively; Cut-off point, sensitivity, specificity and Youden index of PPCP are 0.985, 74.4%, 53.4% and 27.8%, respectively;

After that, we used logistic regression analysis and the results specified that the four parameters performed better in combination for prediction. [AUC = 0.895,95% CI: 0.851– 0.939] (Fig. 5).

## Discussion

Poor glycemic control in patients with type 2 diabetes was reported to be a risk factor for sarcopenia [17]. In our study, we first identified some parameters related to low muscle mass among elderly Chinese participants with T2DM and found that participants with low muscle mass a had significantly lower BMI, BF, ASMI, HOMA-β,ALT,AST, FPCP and PPCP concentrations (*P* < 0.0001 for PPCP; *P* < 0.05 for ALT,AST and FPCP; *P* < 0.001 for BMI, BF, ASMI, and HOMA-β;). In the study conducted by Merchant RA and colleagues, it was indicated that a lower BMI is associated with a higher risk of developing sarcopenia [18]. A previous study conducted on a Chinese female population aged over 50 also indicated that individuals with low muscle mass had lower BMI,ALT levels, consistent with our findings [19]. In a longitudinal cross-sectional study spanning three years in China, it was indicated that there is an association between fasting plasma C-peptide levels and the likelihood of sarcopenia [20]. Inhibition of protein synthesis in patients with diabetic sarcopenia is mediated by inhibition of the IGF1-PI3K-Akt-mTOR pathway [21]. There is evidence to indicate that C-peptide may interact synergistically with the insulin signaling pathway [22]. However, as of now, there is no existing research on the correlation between PPCP and sarcopenia. We are incorporating PPCP into our study to explore potential associations.

Meanwhile, compared with those without low muscle mass, those with low muscle mass had significantly higher female, Ever/current smoking, Ever/current drinking, FPG(*P* < 0.01), HbA1c(*P* < 0.05), and GA(*P* < 0.05) concentration (*P* < 0.001 for gender; *P* = 0.013 for Ever/current smoking; *P* = 0.005 for Ever/current drinking; *P* < 0.01 for FPG; *P* < 0.01 for HbA1c, GA). Some studies indicate a higher prevalence of sarcopenia in elderly male patients with type 2 diabetes mellitus (T2DM), [23, 24] consistent with the findings of our study. Testosterone plays a crucial role in promoting muscle protein synthesis and maintaining muscle mass and function [25]. The skeletal muscle, being the primary organ with glucose transporter 4 (GLUT4), accounts for approximately 80% of the glucose clearance rate [26, 27]. A reduction in muscle mass can induce insulin resistance (IR), leading to compensatory hyperinsulinemia, downregulation of glycogen synthesis, and subsequent elevation of blood glucose levels [28, 29]. Numerous studies have described the vicious effects of smoking on skeletal muscle function and morphology, especially, the thigh muscles [30]. The partial consequence is degeneration in muscle fatigue resistance [31] associated with compromised muscle oxidative capacity [32], and a transition from slow twitch to fast twitch fiber type [33]. Furthermore, smoking could promote skeletal muscle wasting via smoking-induced inflammation that facilitate protein breakdown and suppress protein synthesis [34]. The presence of ethanol has been demonstrated to interfere in muscle with total, microsomal, and mitochondrial protein synthesis, ATPase activity, and substrate oxidation [35–37]. 

We employed Binary Logistic Regression Analysis with stepwise backward deletion to identify the optimal subset that PPCP(OR = 0.497), BMI (OR = 0.548), and female (OR = 0.009) were independent protective factors for low muscle mass in female sarcopenia, but BF(OR = 1.181) was independent risk factors.

Meanwhile, based on the findings of univariate and logistic analysis, we also established ROCs to identify parameters that reflect skeletal muscle mass in the elderly T2DM cohort and found that the AUC of BMI was the highest, followed by PPCP, gender and BF (0.810, 0.675,0.647, and 0.639, respectively), and the AUC of the mixture of the above four reached 0.895.Additionally, we further stratified the analysis by gender, investigating the impact of Skeletal Muscle Mass Index at different sites on glucose metabolism.

In previous studies, patients with sarcopenia exhibited lower insulin levels [10, 19]. In our study, considering the potential impact of insulin therapy on insulin levels, we innovatively utilized C-peptide as a research parameter, circumventing the influence of medication. Our study also indicates lower C-peptide levels in patients with low muscle mass in T2DM. This may be related to the fact that C-peptide exerts an important protective effect against death signaling in myoblasts [38]. We have refined the glucose metabolism in the elderly population with T2DM and have identified a convenient and practical diagnostic tool for assessing low muscle mass. But our study also has limitations, as a cross-sectional study, the causality of this relationship cannot be determined, so further research is needed.

## Conclusion

In this cross-sectional study of elderly Chinese T2DM aged over 60 years, BMI, female and PPCP were associated with T2DM and protective factors for low muscle mass, BF was identified as risk factor for low skeletal muscle.

## Data Availability

The data-set generated and analyzed during the current study will be available from the corresponding author on a reasonable request.

## Abbreviations

T2DM: type 2 diabetes mellitus
